# Linkage Rules for Plant–Pollinator Networks: Trait Complementarity or Exploitation Barriers?

**DOI:** 10.1371/journal.pbio.0050031

**Published:** 2007-01-23

**Authors:** Luis Santamaría, Miguel A Rodríguez-Gironés

**Affiliations:** 1 Mediterranean Institute for Advanced Studies, University of the Balearic Islands/Spanish Council for Scientific Research, Esporles, Mallorca, Spain; 2 Estación Experimental de Zonas Áridas, Spanish Council for Scientific Research, Almería, Spain; University of London, United Kingdom

## Abstract

Recent attempts to examine the biological processes responsible for the general characteristics of mutualistic networks focus on two types of explanations: nonmatching biological attributes of species that prevent the occurrence of certain interactions (“forbidden links”), arising from trait complementarity in mutualist networks (as compared to barriers to exploitation in antagonistic ones), and random interactions among individuals that are proportional to their abundances in the observed community (“neutrality hypothesis”). We explored the consequences that simple linkage rules based on the first two hypotheses (complementarity of traits versus barriers to exploitation) had on the topology of plant–pollination networks. Independent of the linkage rules used, the inclusion of a small set of traits (two to four) sufficed to account for the complex topological patterns observed in real-world networks. Optimal performance was achieved by a “mixed model” that combined rules that link plants and pollinators whose trait ranges overlap (“complementarity models”) and rules that link pollinators to flowers whose traits are below a pollinator-specific barrier value (“barrier models”). Deterrence of floral parasites (barrier model) is therefore at least as important as increasing pollination efficiency (complementarity model) in the evolutionary shaping of plant–pollinator networks.

## Author Summary

Whether they are antagonistic—as between predator and prey—or beneficial—as between pollinator and flower, interactions among all the key species in an ecosystem follow regular patterns. Connectivity (the proportion of possible interactions that are actually realised), for instance, decreases with network size. The “forbidden links” hypothesis proposes that connectivity decreases because interactions are prevented by a mismatch of biological attributes between certain species. Mismatches could arise from the evolution of complementary traits in mutualistic relationships (such as insects preferring to pollinate only flowers of a certain colour) or of traits that prevent exploitation in antagonistic ones (such as a plant growing a long corolla so that insects without a long proboscis cannot reach the nectar reward). We explored the consequences of simple linkage rules based on these two variants on the topology of plant–pollination networks. When compared to data for 37 real plant–pollinator networks, we show that a “mixed” model that combines simple rules from both “complementarity” and “barrier” models best explains the pattern of interactions. This implies, for example, that deterring floral parasites is at least as important as increasing pollination efficiency in the evolution of plant–pollinator networks. Our work emphasises the value of explaining the underlying ecological and evolutionary mechanisms generating such patterns.

## Abstract 3

The topology of plant-pollinator networks can be explained by relatively simple rules incorporating both "complementarity" and "barrier" traits, thus providing insights into the possible evolutionary and ecological processes driving the pattern.

## Introduction

Generalisation is a widespread feature of plant–pollinator interactions [1], and there is a growing interest in the study of complete communities of interacting plants and flower-visiting insects [2,3]. The topology of plant–pollinator qualitative networks (networks that simply consider whether species pairs interact or not) follows certain regular patterns [2–6]. The number of interactions (L) increases with network size (S, the sum of the number of plant species, F, and pollinator species, P), following a power-law relationship [4], while the percentage connectivity [C = 100 × L/(F × P), referred to as connectivity, or C, hereafter] decreases with network size [3]. Furthermore, most plant–pollinator networks are highly nested, i.e., pollinators that visit a plant species are likely to visit more-generalist plant species as well [3,4]. The nestedness (N) of a network increases with its C and size, and it is significantly higher than expected by chance for all moderately large pollination networks (i.e., with more than 50 species) that have been studied [4].

The existing studies on plant–pollinator and seed disperser–plant interactions also suggest that the nested structure of mutualistic networks reflects a fundamental difference from antagonistic webs, arising from how specialisation is distributed among interacting species [4–6]. In contrast to mutualistic networks, antagonistic networks (e.g., predator–prey, herbivore–plant) tend to be more compartmentalised, i.e., characterised by cohesive groups of interacting species with relatively few interactions among groups [7,8]. Several authors have suggested that nested patterns of asymmetrical specialisation may be more likely to develop in mutualistic interactions because natural selection specifically favours the convergence and complementarity of traits in interacting species [9]. In contrast, antagonistic interactions may favour greater compartmentalisation through the continual coevolution of defences and counterdefences (i.e., evolutionary arm races involving exploitation barriers), which generates greater specificity [9].

The idea that nested asymmetries in specialisation are generated by coevolutionary processes has strong links to the concept of “forbidden links” [5]. Under this hypothesis, the topological properties of mutualistic networks result from nonmatching biological attributes of species, such as phenology or morphology, which prevent the occurrence of certain interactions [8]. However, no work to date has explicitly evaluated the extent to which forbidden links can account for network topology, and detailed information about the biology of species interactions is considered a prerequisite for critical tests [10]. The goal of this study is to bridge this gap by considering the extent to which different ecological mechanisms might account for the observed network patterns. Using a set of simple models, we investigated whether linkage rules aimed at representing the two processes outlined above (trait complementarity and exploitation barriers) can explain the topological properties of plant–pollination networks.

We concentrate on two possible mechanisms. In the first mechanism (complementarity traits), the similarity between the reward that the plant has to offer and the resource that the pollinator seeks determines whether species pairs interact. As an example, consider plant and pollinator phenology: a plant will receive visits only of pollinators that are present and active during their flowering period, and plant and pollinator species can become progressively cospecialised by developing increasingly narrower phenological matches [11]. Other examples may include nectar sugar concentration, in which each major pollinator group prefers a specific range of nectar concentrations or sugar compositions [12–15] (although other authors have interpreted nectar dilution as a barrier against bee pollination [16], similar to our second scenario); flower colour, in which flower signals match pollinator perceptual systems (e.g., [17–20], but see also [21,22] for a critical view); and specialised scents [23–25].

In the second scenario (barrier traits), what determines whether species pairs interact is not their similarity but rather the ability of the pollinator to reach the reward offered by the flowers. Each flower type conceals its reward behind barriers, and only those pollinators whose traits allow them to overcome the barriers have access to the reward. As an example, consider the length of hawkmoths' proboscises and corolla tubes [26,27]: hawkmoths can forage efficiently only at flowers with corolla tubes shorter than their proboscises, and it is mainly these flowers that they visit, as shown by Haber and Frankies [28] for a Costa Rican hawkmoth community. Similar examples, involving proboscis or beak length and corolla-tube depth, have been described for bee-, fly-, and hummingbird-pollinated systems [29–33].

To evaluate whether simple linkage rules lead to mutualistic networks with topological properties similar to those observed in actual networks, we used different sets of rules, derived from the stated mechanisms, to simulate the linkage process. We used complementarity and barrier models based on one, two, or four traits and a mixed model based on two complementarity and two barrier traits. For example, in the model with two complementarity and two barrier traits, each individual (plant or pollinator) in a simulated community was assigned two complementarity and two barrier traits at random, and pairs of plants and pollinators interacted if the barrier traits of the pollinator had higher values than the respective traits of the plants and their complementarity traits were “sufficiently” similar. The complementarity models considered three variants (the narrow, medium, and broad complementarity models), depending on how tight the overlap between the plant and pollinator traits had to be for the species to interact. The topology of simulated communities was then compared with the topology of 37 real-world plant–pollinator networks [3,4,34–36], including three unpublished matrices kindly provided by J. M. Olesen. For conciseness, we only describe the results of the models providing a closer fit to the data.

The assumption that the structure of mutualistic networks results from random interactions among individuals can reproduce certain topological properties of these networks [6,10]. To complement our work on mechanistic linkage rules, we used two formulations of the neutrality hypothesis of Vázquez and Aizén [6] to simulate plant–pollinator networks. In these models, the probability that a plant–pollinator pair interacts is proportional to their relative abundance, and relative abundances were drawn from either a uniform (“uniform neutral model”) or a lognormal (“lognormal neutral model”) probability distribution [37,38].

## Results

Most (95%) real-world networks were significantly nested, all exceptions being networks of small size (network size [S] < 50). Most modelled networks were also highly nested, although a few models showed a small proportion (up to 11% for the mixed model) of nonnested networks for small network sizes (S < 160 and, in most cases, S < 70; Table 1).

**Table 1:**
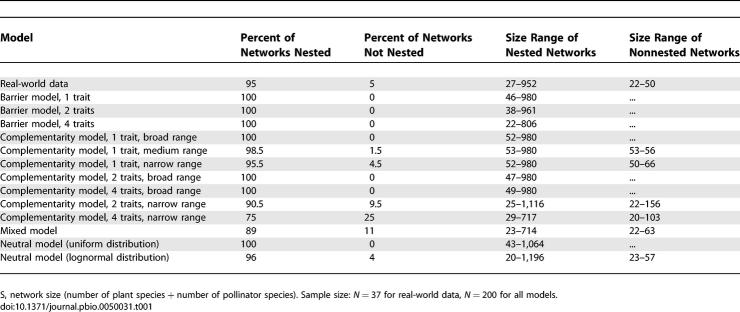
Percentage of Modelled Networks that Were Not Significantly Nested

The scaling properties of networks of increasing species richness (S) differed substantially from those of real-world pollination networks for all one-trait models, most two- and four-trait models, and the uniform neutral model (Table 2; Figures 1 and 2). Only the most restrictive complementarity (two and four traits, narrow range), the four-trait barrier model, the mixed model, and the lognormal-neutral model approached the trends observed in real-world data, and the latter two provided the best fits to most variables.

**Table 2:**
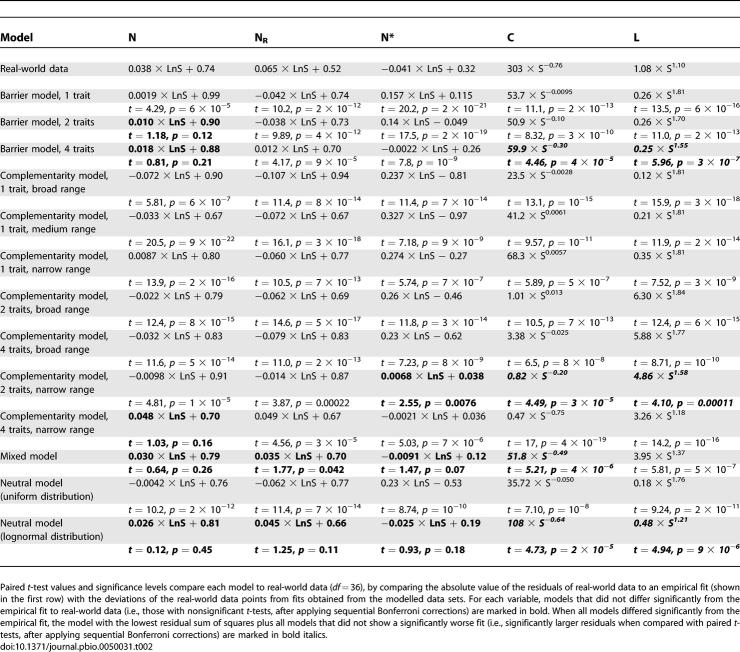
Relationship between Network Topology and Network Size (s)

**Figure 1:**
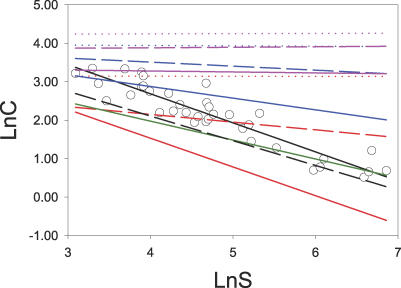
Connectivity of Networks of Increasing Size (S) for Real-World and Modelled Data Matrices Circles indicate real-world data (*N* = 37), the continuous black line indicates an empirical fit to real-world data, and the broken black line is the result of lognormal-neutral models. Colour lines indicate the fits to modelled data sets: narrow-range complementarity models are red, broad-range complementarity models are magenta, barrier models are blue, and mixed models are green. Dotted lines indicate one-trait models, broken lines indicate two-trait models, and continuous lines indicate four-trait models.

**Figure 2:**
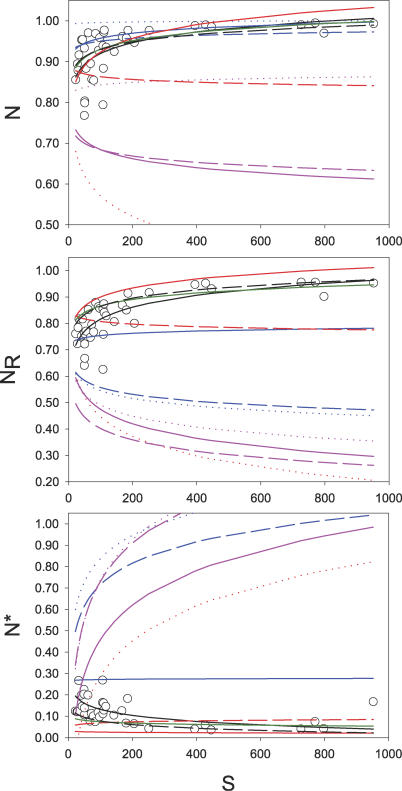
Nestedness of Networks of Increasing Size (S) for Real-World and Modelled Data Matrices Circles indicate real-world data (*N* = 37), the continuous black line indicates an empirical fit to real-world data, and the broken black line is the result of lognormal-neutral models. Colour lines indicate the fits to modelled data sets: narrow-range complementarity models are red, broad-range complementarity models are magenta, barrier models are blue, and mixed models are green. Dotted lines indicate one-trait models, broken lines indicate two-trait models, and continuous lines indicate four-trait models. N_R_ (random matrices). N*, relative nestedness (N/NR)

In real-world networks, the connectivity [C] decreases as a power of S (Figure 1) [3]. While five models (two- and four-trait barrier, four-trait narrow complementarity, mixed, and lognormal-neutral models) approached this behaviour, all of them failed to predict real-world values across the complete range of network sizes and intersected the empirical fit at the lower, medium, and upper part, respectively, of the data range (Figure 1).

The number of interactions (L) increased as a power of S in all models. The exponent of this relationship was greater than the one describing real-world data for all models, and the four-trait barrier, two-trait narrow complementarity, and lognormal-neutral models provided the closest fits (Table 2).

In real-world pollination networks, both the nestedness of the networks (N) and the nestedness of random matrices with similar size and average C (null-model nestedness [N_R_]) increased as the logarithm of S (Table 2; Figure 2). This simultaneous increase of N and N_R_ results in a logarithmic decrease of the relative nestedness [N*]. Five models (two- and four-trait barrier, two-trait narrow complementarity, mixed, and lognormal-neutral models) reproduced remarkably well the logarithmic increase in N with network size, but only two of them (mixed and lognormal-neutral models) performed well in predicting the nestedness of random matrices (N_R_) and, therefore, the N* (Figure 2).

Bascompte et al. [4] divided their mutualistic networks into two groups, according to whether their number of interactions (L) was greater or lesser than expected for their size (positive or negative residuals, respectively, in the regression between L and S). The N* in networks having positive residuals was greater than that in networks with negative residuals (*F* = 6.59, *df* = 1, 50, *p* = 0.013) [4]. This analysis pooled pollination and seed-dispersal networks, and the results did not reach significance when plant–pollinator networks (of which there were 25) were analysed on their own. However, the addition of 12 networks to the data set used here already resulted in a significant difference (*t* = 3.51, *p* = 0.0015, *n* = 37). Running this analysis on the networks generated by the models, we observed that four models (four-trait barrier, two-trait narrow-complementarity, mixed, and lognormal neutral models) produced comparable results (Figure 3).

**Figure 3:**
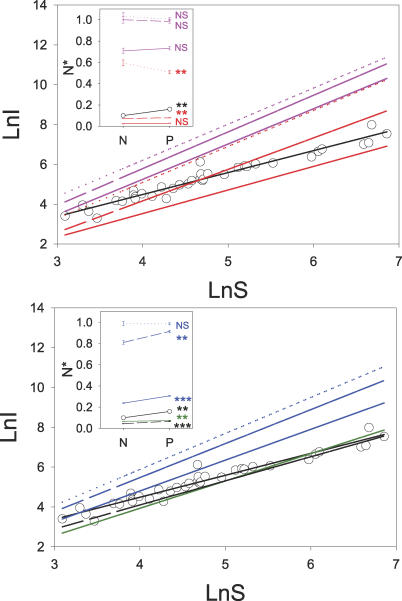
Relationship between Network C and N* for Real-World and Modelled Data Matrices Insets show the N* (average ± SE) of highly and lowly connected communities (i.e., those with positive and negative residuals in the fits of the number of interactions on species richness displayed in the larger panels). Narrow-range complementarity models are red, broad-range complementarity models are magenta, barrier models are blue, and mixed models are green. Dotted lines indicate one-trait models, broken lines indicate two-trait models, and continuous lines indicate four-trait models. Circles indicate real-world data (*N* = 37), the continuous black line indicates an empirical fit to real-world data, and the broken black line is the result of lognormal-neutral models. Asterisks indicate significant differences between highly and lowly connected categories (i.e., those with positive and negative residuals, respectively). *p < 0.05, **p < 0.01, ***p < 0.001, ^NS^ *p* > 0.05.

Because N* decreases with N_R_ and, in the range of interest (C < 50%), N_R_ decreases with C [39], the increased N* of positive L-on-S residuals implies that, as the number of connections of mutualistic networks increases, the nestedness of real-world networks does not decrease as fast as it would in a random matrix. A multiple regression analysis showed that the trends described for real-world data (decreased N and N_R_ and increased N* with increased L, all corrected for the effect of S; Table 3) were shown by the mixed, lognormal-neutral, and four-trait narrow-complementarity models.

**Table 3:**
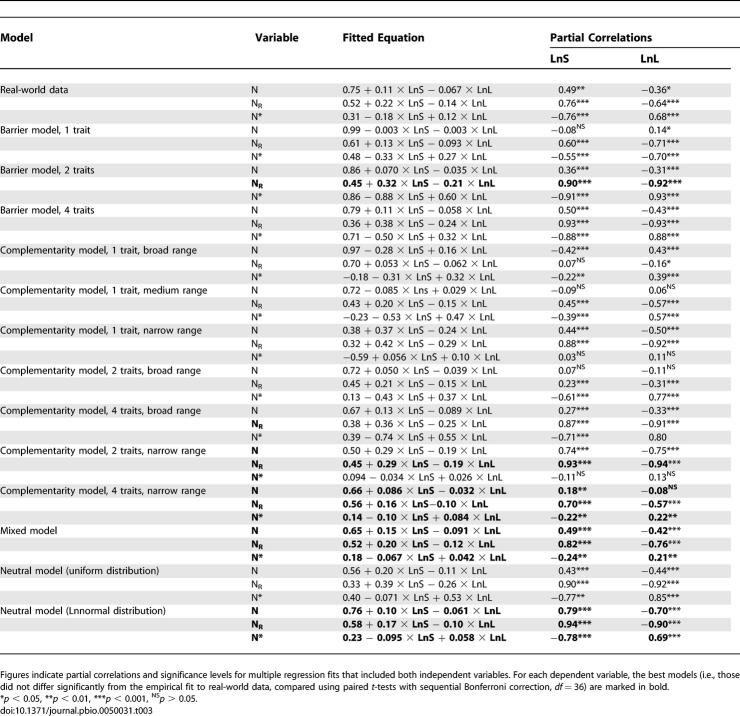
Relationship between Network N, S, and C

## Discussion

Recent work has greatly improved our knowledge of the patterns and scaling laws that characterise plant–pollinator networks, and the current challenge is to understand the ecological and evolutionary processes that underlie these regularities. Our work represents a first step in this direction.

Given the excellent performance of the lognormal neutral model in predicting network characteristics, what is the use of exploring more complex linkage rules? The main purpose of null models is to remind us that showing that a given model fits the data well is not necessarily a demonstration that the model is “correct”: more than one mechanism can produce any given pattern [40]. When two alternatives can explain a certain phenomenon, parsimony demands that we provisionally accept the simpler one, and null models are meant to represent the simplest explanation that can be offered. So, if a null model can be used to explain a pattern, there is no good reason to search for a more complex explanation. There are three reasons, however, why the lognormal neutral model can be rejected as a most-parsimonious explanation of network topology: (1) Assuming random interactions, in itself, is not sufficient to reproduce network topology: the uniform null model provided a very poor fit to the data, and in order to fit the model we had to assume a lognormal distribution of abundances. While the distribution of species abundances in many communities is well described by a lognormal distribution [41], this is an empirical fit. The interpretation of this empirical fit is problematic because it is not clear whether generalist species are generalist because they are more abundant, or they are more abundant because, being generalists, they have access to more resources. (2) The neutral model assumes that species abundance determines the frequency of interactions. This assumption has been recently evaluated by Dupont et al. [36], who found a significant correlation between abundance and generalisation level of the pollinators, but not the plants, in an alpine community. Ollerton et al. [42], on the other hand, reported an association between interaction frequency and generalisation level but found no significant correlation between the relative abundance of insects in the community and their visitation rate to asclepiad plant species. (3) The neutral model assumes that network structure results from random interactions between species and “most phenotypic characteristics of interacting species may be irrelevant in determining broad patterns of interspecific interactions” (e.g., degree distribution) [10]. This assumption is at odds with all reported empirical data, which show that phenotypic traits often prevent the interaction between specific pairs of plants and pollinators [8,33]. For example, Jordano et al. [8] show how 42% of 65% nonrecorded interactions in a plant–hummingbird subnetwork can be attributed to phenological or phenotypic mismatches between the plant species and the pollinator, indicating that a sizeable fraction of the interactions are “forbidden” and thus individuals cannot interact at random. Given that two key assumptions of the neutral model are not supported by empirical data and that its causal interpretation is problematic, and bearing in mind that more than one mechanism can produce any given pattern, we believe that an examination of alternative models based on phenotypic traits is granted.

Let us now turn to the ecological mechanisms (linkage rules) that we have considered. Of these, only the multiple-trait models were compatible with the data, and the combination of both barrier and complementarity rules fitted them best. Although we have tried a number of other mechanistic models, such as stochastic versions of the complementarity and barrier models, and models that combined a lower amount of complementarity and barrier traits (not dealt with in depth for the sake of conciseness), we have been unable to produce any version that fitted the data better than the mixed model presented here.

Although the optimal fit of the four-trait mixed model does not necessarily imply that it provides a realistic description of the linkage rules responsible for network assembly in the real world [40], we have shown that simple ecological processes may well lie behind the complexity of these large networks, and this result should encourage us to search for linkage rules in the field. A minimum amount of complexity is nevertheless required to explain real-world networks, since a combination of at least four traits was necessary to reproduce the patterns observed there.

The poor fit of the complementarity models to the data shows that the complementarity rule, in itself, cannot be the linkage rule we are looking for. It should be noted that this result can be taken at face value: a model providing a poor fit to the data can be dismissed regardless of whether a better model is known. The complementary and barrier traits had contrasting effects on network characteristics. Plant–pollinator pairs specialised on each other predominate with complementarity models, leading to highly connected networks of low nestedness. To approach the nestedness of real-world networks, we must impose very restrictive conditions (narrow ranges with several traits), and under these conditions, networks are very sensitive to random effects (high values of N_R_) and show excessively low C. With barrier models, on the other hand, specialised plants interact with pollinators that have access to very diverse resources, producing highly nested networks. In mixed models, which provide the best fits, complementary traits relax the trend to excessive nestedness of barrier models, and barrier models relax the too-low connectance and the high dependence of random effects of complementary traits.

The demonstration that the complementary rule alone is unable to produce realistic network topologies has important evolutionary implications. It suggests that nested patterns of asymmetrical specialisation observed in mutualistic interactions do not arise because natural selection on mutualisms specifically favours the convergence and complementarity of traits in interacting species. Plant–pollinator coevolution may be the result of selection for plant traits that enhance visitation rates by the most efficient pollinator and/or pollination efficiency by the most common pollinator [1,17,43], but floral evolution might also represent a compromise between attraction and defence [44–48]. Although this latter hypothesis has been questioned because deterrent traits will interfere with nectar exploitation by efficient pollinators [49], recent models incorporating foraging decisions have shown that the evolution of pollinator-deterring traits is possible, provided that deterring parasites (i.e., floral visitors that, by reducing pollen availability to more-efficient pollinators, decrease the plant's fitness) is sufficiently beneficial [50]. Facilitation of most-efficient pollinators predicts an increasing narrowing of the ecological range of plants and pollinators as a consequence of coevolution. In such a scenario, evolution leads to an increasingly tighter match of the coevolved morphological structures (and/or functional and behavioural traits) of plants and animals: the complementarity rule. In contrast, parasite deterrence leads to the evolution of floral barriers and pollinator structures allowing them to overcome plant defences: the barrier rule. The inability of the complementarity rule to mimic the topology of real-world networks thus suggests that the most-efficient–pollinator principle is not the main (or the exclusive) driving force behind the evolution of floral and pollinator traits. The optimal performance of the mixed model indeed suggests that both the most-efficient–pollinator principle and parasite deterrence operate simultaneously as evolutionary forces in natural communities.

Barrier traits also provide an answer to Vázquez's main criticism of Jordano's forbidden interactions: “forbidden links resulting from phenological or morphological constrains are equally likely to affect any species, not just the most connected ones, and it is unclear whether this assembly constraint would necessarily lead to a decay in the tail of the degree distribution” [10]. By its very nature, barrier traits affect specialist and generalist, plant and insect species asymmetrically. While large trait values result in ecological specialisation for plant species (i.e., few insects have trait values large enough to access their resources), they result in ecological generalisation for insect species (i.e., insects with large trait values have access to many plant species). The asymmetrical effect of large trait values on plants and insects automatically generates nested patterns in the interaction networks. More important, competition for floral resources results in niche segregation that favours the exploitation of specialised (i.e., large trait) plants by generalist (i.e., large trait) insects, and vice versa [50], thereby imposing an upper limit to the number of interactions that a generalist can sustain at any given time.

The nested pattern seems to be a pervasive feature of mutualistic networks and has also been found in seed-dispersal networks and, more recently, in plant–ant [9] and fish–anemone [51] communities. Our results suggest that these systems may also be characterised by the coexistence of both complementarity and barrier traits. Examples of both types of traits for seed-dispersal networks may respectively include fruiting phenology and disperser availability, on the one hand, and fruit size and gape width, on the other [52]. Nevertheless, it must be noted that our work studies, other than network nestedness, the relationships between network size and various topological properties (L, C, N, N_R_, and N*). In order to explore the applicability of complementarity and barrier linkage rules to other mutualistic systems, we would need to know how the topological properties of these networks scale with size.

Our study represents a first effort to go beyond the description of network topology and into the analysis of the ecological and evolutionary processes behind it, which may complement (and aid the interpretation of) other attempts based on empirical studies [8,36,42]. The results presented in this paper stress the utility of mechanistic, phenotype-based approaches to community-level questions. To elucidate the actual ecological processes responsible for the assembly of plant–pollinator networks, we need detailed quantitative descriptions of the plant–pollinator networks, the relationship between traits of interacting plant–pollinator pairs, and the implications of phenotypic traits for plant and pollinator fitness.

## Materials and Methods

We restrict our analysis and discussion to qualitative networks. Plant–pollinator networks are characterised by their interaction matrix, in which rows represent pollinator species and columns represent plant species. The *ij* cell of the matrix is set to “1” if pollinator species *i* interacts with plant species *j*, and to “0” otherwise.

To evaluate whether simple linkage rules lead to mutualistic networks with the same topology as the networks actually observed, we used different sets of rules to simulate the linkage process and compared the topology of simulated communities with the topology of 37 real-world plant–pollinator networks [3,4,34–36], plus three unpublished matrices kindly provided by J. M. Olesen.

For each model, we simulated 200 communities of different sizes by selecting a random number of potential plant species (F; in the range of 10 to 160, uniform distribution), letting the number of potential pollinators be P = 45.573 − 0.8082 × F + 0.047 × F^2^ (this regression was derived from the real-world communities considered in the study) and assigning traits at random to plants and pollinators to determine which pairs interacted (as determined below for each particular model). We then removed all plants and pollinators that had no interactions, arriving at F_1_ and P_1_ interacting plants and pollinating species, respectively. Relationships between network topology and community size are all based on the value of S_1_ = F_1_ + P_1_. Network topology was described by the total L, C, N, and N* [4,5]. A mutualistic network is perfectly nested if each plant species is visited by a subset of the pollinators visiting more-generalist plants [4,5], the “temperature” (T) of the network is a normalised measure of the network's deviation from perfect nestedness [4,5] and ranges from 0 to 100, and the level of nestedness is defined as N = (100 – T)/100 and ranges from 0 to 1 [4]. For each community that we simulated, we used 100 random matrices to assess the extent to which the level of nestedness of the hypothetical community deviated from random expectations and to calculate N*: N* = (N – N_R_)/N_R_, where N_R_ is the average nestedness of the random replicates (“null-model nestedness”). The random replicates were generated using the most conservative null model (null model 2) proposed by Bascompte et al. [4].

Interaction matrices were generated with a C++ program, available upon request, as detailed below for each model. All “random” numbers were generated with the function “ran1” from [53]. The program calculates nestedness using BINMATNEST (available at http://www.eeza.csic.es/eeza/personales/rgirones.aspx [39]), an improved version of the Nestedness Temperature Calculator [54,55].

### Single-trait complementarity model.

The single-trait complementarity model assumes that plants and pollinators can be described by a single trait. Each species is characterised by a mean trait value and a range of variability, and a pair of species will interact if their traits overlap. Let *V_i_* and *W_j_* be the central trait value for pollinator species *i* and plant species *j,* respectively, and let δ*V_i_* and δ*W_j_* be their ranges of variability. Then, the value of the interaction matrix corresponding to this pair of species, *I_ij_,* will be

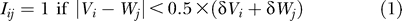
and

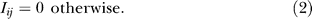
We considered three scenarios (hereafter referred to as broad-, medium-, and narrow-range complementarity models, respectively) that differed in the average value of δ*V_i_* and δ*W_j_*. In all cases, the *V_i_* and *W_j_* were independent random variates with uniform distribution in the interval (0, 1). The δ*V_i_* and δ*W_j_,* on the other hand, were random variates with uniform distributions in the intervals (0, 1), (0, 0.5), and (0, 0.25), for the broad-, medium-, and narrow-range complementarity models, respectively.

For many traits, there is a correlation between average value and variability (J. M. Olesen, unpublished data). Including this correlation in the model lead to identical results to the ones with uncorrelated mean and variability, and the results will not be discussed further.

### Two- and four-trait complementarity models.

In most, if not all, real communities, flowers differ along several dimensions. The two- and four-trait complementarity models consider the possibility that pollinators must fit several floral traits in order to reach the reward offered by flowers. The flowers of the *j*th plant species are thus characterised by *N* central trait values and ranges of variability (*N* = 2 or 4 for the two- and four-trait complementarity models, respectively), *W_j_^k^* and δ*W_j_* ^*k*^, with *k* = 1,… *N.* Similarly, the pollinators of the *i*th species are characterised by central trait values *V_i_^k^* and ranges of variability δ*V_i_* ^*k*^, with *k* = 1,… *N.* Central trait values for plants and pollinators were independent random variates with uniform distribution in (0, 1), and ranges of variability were independent random variates with uniform distribution in the range (0, 1), (0, 0.5), or (0, 0.25) for the broad-, medium-, and narrow-range complementarity models, respectively. The interaction matrix is given by

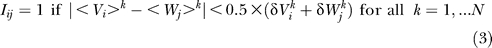
and

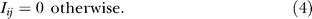



### One-trait barrier model.

Like the complementarity model, the one-trait barrier model assumes that flowers and pollinators can be described by a single trait. We ignore variability in this case and simply assume that pollinators of species *i* will visit flowers of species *j* if their trait, *V_i_,* is greater than the barrier of the plant, *W_j_* (assuming that pollinators of species *i* will visit flowers of species *j* if *V_i_* < *W_j_* is mathematically equivalent and leads to exactly the same results). As a result, the interaction matrix takes the values

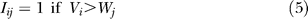
and

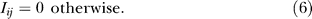



The *V_i_* and *W_j_* were independent random variates with uniform distributions in the interval (0, 1).

### Two- and four-trait barrier models.

The two- and four-trait barrier models consider the possibility that pollinators must overcome several barriers in order to reach the reward offered by flowers. The flowers of the *j*th plant species are thus characterised by *N* barriers (*N* = 2 or 4 for the two- and four-trait barrier models, respectively), *W_j_^k^,* with *k* = 1,… *N*, and the pollinators of the *i*th species are characterised by traits *V_i_^k^,* with *k* = 1,… *N,* where the *V_i_^k^* and *W_j_^k^* are independent random variates with uniform distribution in (0, 1). The interaction matrix is given by

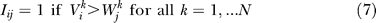
and

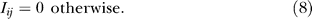



### Mixed models.

In real communities, the interactions between flowers and pollinators are likely to combine both complementarity and barrier types of traits (J. M. Olesen, M. Price, and N. Waser, unpublished data). The mixed models combine complementarity and barrier types of traits (two traits each). For this purpose, we selected the barrier model that produced the best fit to real-world data: the narrow-complementarity model.

The flowers of the *j*th plant species are thus characterised by two barrier traits, *W_j_^k^,* two ranges of variability δ*W_j_* ^*k*^ (*k* = 1, 2), and two central trait values, *W_j_^k^* (*k* = 3, 4). The pollinators of the *i*th species are characterised by two barrier traits, *V_i_^k^,* two ranges of variability δ*V_i_* ^*k*^ (*k* = 1, 2), and two central trait values, *V_i_^k^* (*k* = 3, 4). All variables are independent random variates with uniform distribution and ranges (0, 1) for the barrier traits and central trait values and (0, 0.25) for the ranges of variability. The interaction matrix is given by

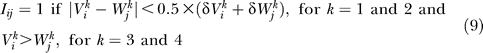
and

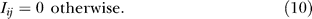



### Neutral model.

The neutral models assume that interactions between plants and pollinators are determined by the relative abundance of the species. The *j*th plant species is characterised by its relative abundance, *W_j_,* and the pollinators of the *i*th species by their relative abundance, *V_i_,* where the *V_i_* and *W_j_* are independent random variates with uniform distribution in (0, 1). The interaction matrix is given by

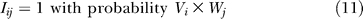
and

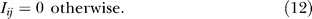



### Lognormal neutral model.

The lognormal neutral model was similar to the neutral model, except that the *V_i_* and *W_j_* are now independent random variates with lognormal distribution. The interaction matrix is given by

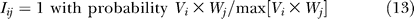
and

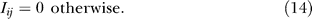



We studied 200 communities generated with the algorithm specified above and used the same parameters to analyse the topology of the networks generated.

### Comparisons among models.

Models were compared on the basis of their fit to real-world data. We first fitted separate regression lines (logarithmic for N, N_R_, and N*, power fit for C and I) to each data set generated by the various model (model fits). For each model, we then calculated the deviation of the real-world data points from the model fit (i.e., the absolute value of the difference between each real-world data point and the expected value, calculated using the model fit) and compared these deviations with the absolute value of the residuals of the real-world data points to a regression line fitted through these points (same type of regression as above: logarithmic for N, N_R_, and N*, power fit for C and I). Deviations from model fits and residuals were compared using paired *t*-tests with sequential Bonferroni correction. We used paired *t*-tests because we compared pairs of distances for each data point: the deviation from the model fit versus the absolute value of the residual from the empirical fit. We considered as best models all those that did not differ significantly from the empirical fit (i.e., those which do not perform significantly worse than an empirical fit). Whenever all models performed significantly worse than the empirical fits (i.e., for C and I), we performed multiple comparisons among all models using paired *t*-tests (similar to above but comparing for each real-world data point the deviation from one model fit versus the deviation from the other model fit) with sequential Bonferroni correction. We considered as best models those with the best fit to real-world data (i.e., the lowest sum of squared deviations) plus all those that did not perform significantly worse (i.e., those that did not show significantly higher residuals) than them.

## Abbreviations

*C*: connectivity
F: number of plant species
L: number of interactions
N: nestedness
N*: relative nestedness
N_R_: null-model nestedness
P: number of pollinator species
S: network size (number of species)
T: temperature
